# Holmium-166 radioembolization for the treatment of patients with liver metastases: design of the phase I HEPAR trial

**DOI:** 10.1186/1756-9966-29-70

**Published:** 2010-06-15

**Authors:** Maarten LJ Smits, Johannes FW Nijsen, Maurice AAJ van den Bosch, Marnix GEH Lam, Maarten AD Vente, Julia E Huijbregts, Alfred D van het Schip, Mattijs Elschot, Wouter Bult, Hugo WAM de Jong, Pieter CW Meulenhoff, Bernard A Zonnenberg

**Affiliations:** 1 Department of Radiology and Nuclear Medicine, University Medical Center Utrecht, Heidelberglaan 100, E01.132, 3584 CX Utrecht, The Netherlands; 2 Department of Clinical Pharmacy, University Medical Center Utrecht, Heidelberglaan 100, 3584 CX Utrecht, The Netherlands

## Abstract

**Background:**

Intra-arterial radioembolization with yttrium-90 microspheres ( ^90^Y-RE) is an increasingly used therapy for patients with unresectable liver malignancies. Over the last decade, radioactive holmium-166 poly(L-lactic acid) microspheres ( ^166^Ho-PLLA-MS) have been developed as a possible alternative to ^90^Y-RE. Next to high-energy beta-radiation, ^166^Ho also emits gamma-radiation, which allows for imaging by gamma scintigraphy. In addition, Ho is a highly paramagnetic element and can therefore be visualized by MRI. These imaging modalities are useful for assessment of the biodistribution, and allow dosimetry through quantitative analysis of the scintigraphic and MR images. Previous studies have demonstrated the safety of ^166^Ho-PLLA-MS radioembolization ( ^166^Ho-RE) in animals. The aim of this phase I trial is to assess the safety and toxicity profile of ^166^Ho-RE in patients with liver metastases.

**Methods:**

The HEPAR study (Holmium Embolization Particles for Arterial Radiotherapy) is a non-randomized, open label, safety study. We aim to include 15 to 24 patients with liver metastases of any origin, who have chemotherapy-refractory disease and who are not amenable to surgical resection. Prior to treatment, in addition to the standard technetium-99m labelled macroaggregated albumin ( ^99m^Tc-MAA) dose, a low radioactive safety dose of 60-mg ^166^Ho-PLLA-MS will be administered. Patients are treated in 4 cohorts of 3-6 patients, according to a standard dose escalation protocol (20 Gy, 40 Gy, 60 Gy, and 80 Gy, respectively). The primary objective will be to establish the maximum tolerated radiation dose of ^166^Ho-PLLA-MS. Secondary objectives are to assess tumour response, biodistribution, performance status, quality of life, and to compare the ^166^Ho-PLLA-MS safety dose and the ^99m^Tc-MAA dose distributions with respect to the ability to accurately predict microsphere distribution.

**Discussion:**

This will be the first clinical study on ^166^Ho-RE. Based on preclinical studies, it is expected that ^166^Ho-RE has a safety and toxicity profile comparable to that of ^90^Y-RE. The biochemical and radionuclide characteristics of ^166^Ho-PLLA-MS that enable accurate dosimetry calculations and biodistribution assessment may however improve the overall safety of the procedure.

**Trial registration:**

ClinicalTrials.gov NCT01031784

## Background

The liver is a common site of metastatic disease. Hepatic metastases can originate from a wide range of primary tumours (e.g. colorectal-, breast- and neuroendocrine tumours) [1]. It is estimated that 50% of all patients with a primary colorectal tumour will in due course develop hepatic metastases [2]. Once a primary malignancy has spread to the liver, the prognosis of many of these patients deteriorates significantly. Potentially curative treatment options for hepatic metastases consist of subtotal hepatectomy or, in certain cases, radiofrequency ablation. Unfortunately, only 20-30% of patients are eligible for these potentially curative treatment options, mainly because hepatic metastases are often multiple and in an advanced stage at the time of presentation [3]. The majority of patients are therefore left with palliative treatment options.

Palliative therapy consists primarily of systemic chemotherapy. In spite of the many promising developments on cytostatic and targeted biological agents over the last ten years, there are still certain tumour types that do not respond adequately and the long-term survival rate for patients with unresectable metastatic liver disease remains low [4-8]. Moreover, systemic chemotherapy can be associated with substantial side effects that lie in the non-specific nature of this treatment. Cytostatic agents are distributed over the entire body, destroying cells that divide rapidly, both tumour cells and healthy cells. For these reasons, a significant need for new treatment options is recognized.

A relatively recently developed therapy for primary and secondary liver cancer is radioembolization with yttrium-90 microspheres ( ^90^Y-RE). ^90^Y-RE is a minimally invasive procedure during which radioactive microspheres are instilled selectively into the hepatic artery using a catheter. The high-energy beta-radiation emitting microspheres subsequently strand in the arterioles (mainly) of the tumour, and a tumoricidal radiation absorbed dose is delivered. The clinical results of this form of internal radiation therapy are promising [9,10]. The only currently clinically available microspheres for radioembolization loaded with ^90^Y are made of either glass (TheraSphere ^®^, MDS Nordion Inc., Kanata, Ontario Canada) or resin (SIR-Spheres ^®^, SIRTeX Medical Ltd., Sydney, New South Wales, Australia).

Although ^90^Y-RE is evermore used and considered a safe and effective treatment, ^90^Y-MS have a drawback: following administration the actual biodistribution cannot be accurately visualized. For this reason, holmium-166 loaded poly(L-lactic acid) microspheres ( ^166^Ho-PLLA-MS) have been developed at our centre [11,12]. Like ^90^Y, ^166^Ho emits high-energy beta particles to eradicate tumour cells but ^166^Ho also emits low-energy (81 keV) gamma photons which allows for nuclear imaging. As a consequence, visualization of the microspheres is feasible. This is very useful for three main reasons. Firstly, prior to administration of the treatment dose, a small scout dose of ^166^Ho-PLLA-MS can be administered for prediction of the distribution of the treatment dose. This provides a theoretical advantage over ^90^Y-RE, for which the distribution assessment depends on a scout dose of ^99m^Tc-MAA, with a disputable distribution correlation with the actual microspheres [13]. Secondly, quantitative analysis of the nuclear images would allow assessment of the radiation dose delivered on both the tumour and the normal liver (i.e. dosimetry) [14]. Thirdly, since holmium is highly paramagnetic, it can be visualized using magnetic resonance imaging (MRI). Quantitative analysis of these MRI images is also possible, which is especially useful for medium- and long-term monitoring of the intrahepatic behaviour of the microspheres [15,16].

The pharmaceutical quality of ^166^Ho-PLLA-MS has been thoroughly investigated and proven to be satisfactory [17-19]. Multiple animal studies have been conducted in order to investigate the intrahepatic distribution (ratio tumour to normal liver), the toxicity profile/biocompatibility of the ^166^Ho-PLLA-MS, safety of the administration procedure, and efficacy of these particles [20-23].

Now that the preclinical phase of ^166^Ho-RE has been successfully completed, we will start a clinical trial (the HEPAR study: **H**olmium **E**mbolization **P**articles for **A**rterial **R**adiotherapy) in order to evaluate ^166^Ho-RE in patients with liver metastases. The main purpose of this trial is to assess the safety and toxicity profile of ^166^Ho-RE. Secondary endpoints are tumour response, biodistribution prediction with ^99m^Tc-MAA versus a safety dose of ^166^Ho-PLLA-MS, performance status, and quality of life.

## Methods

### Study design

The HEPAR study is a single centre, non-randomized, open label safety study. In this phase I study, a new device will be investigated, namely ^166^Ho-PLLA-MS for intra-arterial radioembolisation for the treatment of liver malignancies. In a group of 15 to 24 patients with liver metastases, treated with increasing amounts of ^166^Ho, the device will be investigated for safety and toxicity.

### Subjects

The study will include patients with liver-dominant metastases, of any histology, who cannot be treated by standard treatment options such as surgery and systemic chemotherapy, due to advanced stage of disease, significant side effects or unsatisfactory tumour response. The detailed inclusion and exclusion criteria are listed in Appendix 1.

### Time schedule

Patient recruitment will take place between October 2009 and January 2011.

### Medical device

**Figure 1:**
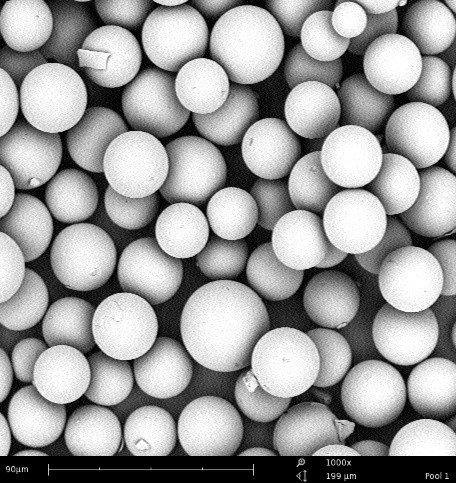
**Scanning electron microscope image of holmium microspheres**.

Using the solvent evaporation technique, non-radioactive holmium-165 ( ^165^Ho) and its acetylacetonate complex (HoAcAc) can be incorporated into the poly(L-lactic acid) matrix to form microspheres (Figure 1). Subsequently, the non-radioactive ^165^Ho-PLLA-MS can be made radioactive by neutron activation in a nuclear facility and form ^166^Ho-PLLA-MS. Neutron-activated ^166^Ho has a half-life of 26.8 hours and is a beta emitter (E_βmax _= 1.85 MeV) that also emits gamma photons (E_γ _= 81 keV) suitable for single photon emission computed tomography (SPECT) (Table 1).

**Table 1:** Microsphere characteristics

Microsphere type	Ho-PLLA-MS	TheraSphere^®^	SIR-Spheres^®^
Matrix material	PLLA	Glass	Resin
Isotope	^166^Ho	^90^Y	
Physical half-life (h)	26.8	64.1	
Υ-energy (keV)	81	no Υ-emission	
β-energy (MeV)	1.77 (48.7%) 1.85 (50.0%)	2.28 (99.9%)	
Neutron absorption cross-section (barn)	64	1.3	
Activity/sphere (Bq)	≤ 450	2500	50
*n *particles instilled	33 million	4 million	50 million
Density (g/ml)	1.4	3.3	1.6

### Recruitment

Patients with liver metastases who agree to participate in the study must be referred to the principle investigator by the department of Surgery. The principle investigator will inform every patient and obtain their informed consent.

### Pre-treatment work-up

#### Screening

A screening visit will take place at the outpatient clinic within 14 days prior to the fist angiography. During this visit, the principle investigator will run through the inclusion and exclusion criteria, conduct a physical examination, and assess the WHO performance status of the patient. Subsequently, CT, MRI, and positron emission tomography (PET) will be performed, as well as electrocardiography (ECG). PET will only be performed in FDG-avid tumours. Liver weight will be calculated, based on the liver volume measured on CT data with a density conversion factor of 1.0 g/cm ^3^. Relevant laboratory tests (haematology, coagulation profile, serum chemistry, tumour marker) must be documented and reviewed. All patients are asked to fill out the European Organisation for Research and Treatment of Cancer (EORTC) QLQ-C30 questionnaire [24].

#### Angiography

Patients will be hospitalized on the evening prior to angiography. On day 0 the patient is subjected to angiography of the upper abdominal vessels. The celiac axis and superior mesenteric artery are visualised, followed by coiling of relevant vessels, in particular branches of the hepatic artery supplying organs other than the liver, e.g. gastroduodenal artery (GDA), right gastric artery (RGA). If major arteries like the GDA or RGA cannot be successfully occluded, the patient will be withheld ^166^Ho-RE. This procedure will be performed by a skilled and trained interventional radiologist. The catheter is introduced using the Seldinger technique. Prior to the procedure, the patient is offered a tranquilizer (oxazepam 1 dd 10 mg). Premedication consists of a single administration of corticosteroids (dexamethason 10 mg i.v.) and antiemetics (ondansetron 8 mg i.v.). Proton pump inhibitors (pantoprazol 1 dd 40 mg) are started on the day of the intervention and prescribed for use until the end of the follow-up.

#### Macroaggregated albumin injection

After successful angiography and coiling of relevant vasculature is performed, a dose of ^99m^Tc-Macroaggregated Albumin ( ^99m^Tc-MAA) will be administered in the hepatic artery on the same day. The ^99m^Tc-MAA are used to assess whether a favourable distribution of the ^166^Ho-PLLA-MS can be expected. The patient is subjected to planar imaging of the thorax and abdomen and SPECT of the abdomen, in order to determine the ^99m^Tc-MAA distribution. Images will be evaluated qualitatively and quantitatively. Extrahepatic deposition of activity is a contra-indication for administration of the treatment dose. Region of interest analysis will be used to calculate lung shunting. Lung shunting should not exceed 20% of the dose ^99m^Tc-MAA. If the amount of lung shunting cannot be reduced to <20% using standard radiological interventional techniques to decrease the shunting, the patient will not be eligible to receive a safety nor a treatment dose of ^166^Ho-PLLA-MS. The dose point-kernel method will be applied to the (non-homogeneous) activity distribution to calculate the absorbed dose distribution [25]. Dose-volume histograms will be generated in order to quantify the dose distribution, and the tumour to healthy tissue absorbed dose ratio will be calculated.

#### ^166^Ho-PLLA-MS safety dose

The second angiography takes place around 1 week after the first angiography but no longer than 2 weeks later. Patients will be hospitalized on the evening before the day of treatment. They will be discharged approximately 48 hours after the intervention unless complications have occurred. Prior to the procedure, the patient is offered a tranquilizer (oxazepam 10 mg)**. **A safety dose of ^166^Ho-PLLA-MS will be administered through a catheter inside the hepatic artery, at the position planned during the first intervention. The safety dose will consist of 60 mg (10% of the total amount of microspheres) ^166^HoPLLA-MS with a lower specific activity (90 Bq/microsphere) than for the treatment dose. After the safety dose, planar imaging of both the thorax and abdomen will be performed, as well as SPECT and MRI of the abdomen. Presence of inadvertent administration to the lungs or other upper abdominal organs will once more be checked for. These SPECT and MRI images will be compared with the images post ^99m^Tc-MAA and post-treatment, regarding extrahepatic deposition of activity, percentage lung shunting, homogeneity of the dose distribution and tumour to healthy tissue absorbed dose ratio.

### Treatment

#### ^166^Ho-PLLA-MS treatment dose

**Figure 2:**
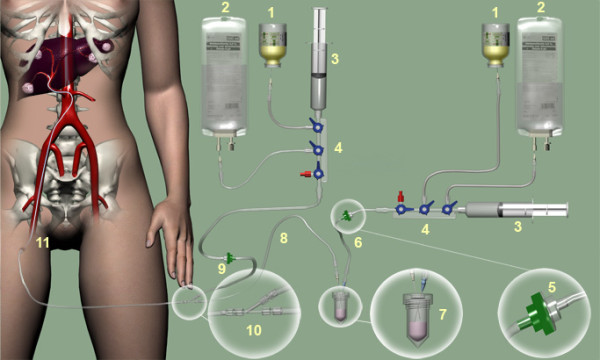
**Schematic overview of the administration system for ^166^Ho-RE**.The administration system consists of the following components: iodine contrast agent (Visipaque ^®^, GE Healthcare) (1), saline solution (2), 20-ml syringe (Luer-Lock) (3), three-stopcock manifold (4), one-way valve (5), inlet line (6), administration vial containing the ^166^Ho-PLLA-MS (7), outlet line (8), flushing line (9), Y-connector (10) and catheter (11).

When the amount of lung shunting does not exceed 20% of the safety dose of ^166^HoPLLA-MS, the (complete) treatment dose of ^166^HoPLLA-MS will be administered (Figure 2). Consecutive cohorts of 3 patients will be treated with identical amounts of microspheres (600 mg), and the last cohort will consist of at least 6 patients. If no toxicity ≥ grade 3 according to the Common Terminology Criteria for Adverse Events (CTCAE)[26] is observed, the next cohort of three patients will be treated at the next radiation dose level. If in one patient CTCAE ≥ grade 3 is observed in a particular cohort, the cohort will be extended to six patients. If toxicity ≥ grade 3 is observed in two or more patients in a particular cohort, the study will be terminated because the endpoint, e.g. the maximum tolerated radiation dose, is reached. This will be reported to the Independent Ethics Committee (IEC). The dose level prior to the toxic radiation dose will become the recommended dose for efficacy studies. If an event is classified as grade 3 or 4 administration technique related, the patient will be replaced. The specific activity of the ^166^Ho-PLLA-MS will be increased by adapting the activation time in the nuclear reactor. The first, second, third and fourth cohort will be treated with a dose of 1.3, 2.5, 3.8 and 5.0 GBq/kg (liver weight), respectively. Assuming a homogenous uptake throughout the liver, this equals escalating radiation doses of 20 Gy, 40 Gy and 60 Gy, to a maximum dose of 80 Gy in the last cohort. A maximum of 15.1 GBq will be given to the maximum treated liver weight (inclusive the tumour tissue) of 3 kg (Table 2). The amount of radioactivity administered to the patient is calculated according to the following formula:

**Table 2:** Dose (Gy) and activity (MBq) relation of ^166^Ho treatment

	Liver weight (kg)				
	1	1,5	2	2,5	3
Liver dose (Gy)	A (MBq)	A (MBq)	A (MBq)	A (MBq)	A (MBq)

10	630	945	1260	1575	1890
**20**	**1260**	**1890**	**2520**	**3150**	**3780**
30	1890	2835	3780	4725	5670
**40**	**2520**	**3780**	**5040**	**6300**	**7560**
50	3150	4725	6300	7875	9450
**60**	**3780**	**5670**	**7560**	**9450**	**11340**
70	4410	6615	8820	11025	13230
**80**	**5040**	**7560**	**10080**	**12600**	**15120**

In bold: the four consecutive cohorts receive 1.3 GBq/kg (20 Gy), 2.5 GBq/kg (40 Gy), 3.8 GBq/kg (60 Gy) and 5.0 GBq/kg (80 Gy), respectively. As an example, a patient in the first cohort (20 Gy) with a 1.5-kg liver, will be administered a total activity of 1890 MBq

where LW is the liver weight of the patient which may be determined using CT, MRI or ultrasound, and where 15.87 × 10 ^-3 ^(J/MBq) is the activity-to-dose conversion factor for ^166^Ho [23].

### Radiation exposure rate

During the hospitalization in week 1 the radiation exposure rate will be measured from 1 m distance at t = 0, 3, 6, 24, and 48 hours following ^166^Ho-PLLA-MS administration. Patients will not be discharged from the hospital until the dose equivalent is less than 90 μSv/h measured from 1 m distance.

### Follow-up

**Figure 3:**
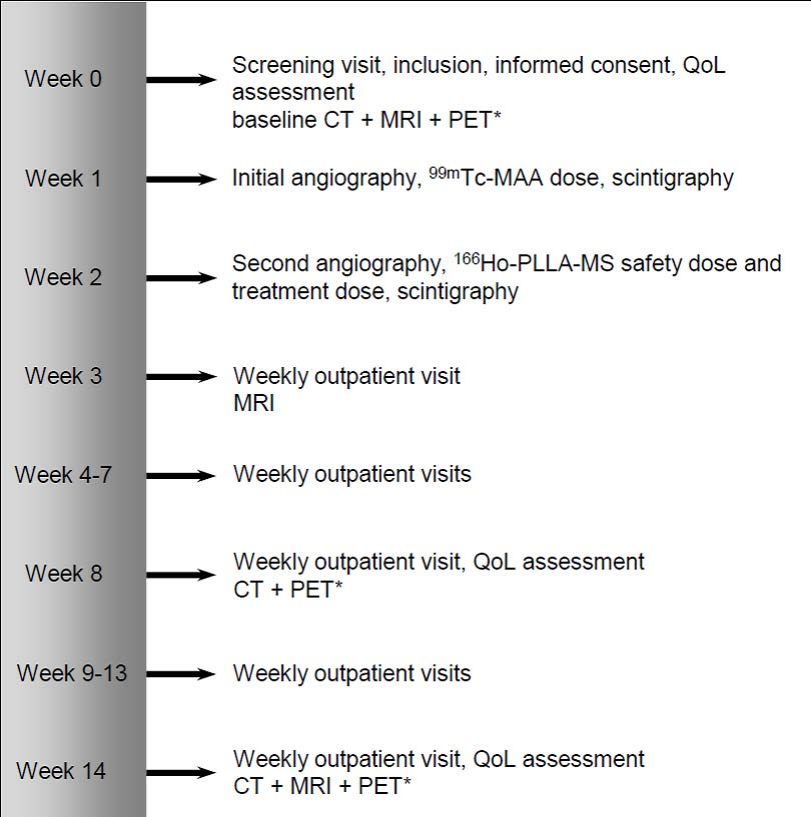
**Timeline for study participants**. *only in ^18^F-FDG-avid tumours.

All patients are followed over a period of 12 weeks after treatment with weekly visits at the outpatient clinic. During each visit, data is collected by physical examination, WHO performance status assessment and laboratory examination (haematology, coagulation profile, serum chemistry and (if applicable) tumour marker). Adverse events are monitored. In addition, patients are asked to fill out the EORTC questionnaires in the 6 ^th ^and 12 ^th ^week post-treatment. CT and (in case of ^18^F-FDG-avid tumours) PET are performed in the 6 ^th ^and 12 ^th ^week post-treatment and MRI is performed in the 1 ^st ^and the 12 ^th ^week post-treatment (Figure 3).

#### Holmium content

Pooled urine samples will be collected from 0-3 hours, 3-6 hours, 6-24 hours and 24-48 hours post- ^166^Ho-PLLA-MS administration. In the 6 ^th ^and 12 ^th ^week post treatment, pooled 24-hours urine will be collected for measurement of holmium content. The date and time of the start and the end of the collection period, the volume and whether the collection was complete or not, will be noted in the case record form. During the hospitalization in week 1, blood will be drawn for measuring the holmium content in the blood at t = 0, 3, 6, 24, and 48 hours following ^166^Ho-PLLA-MS administration. Measurements will be done according to activity measurement of holmium-166 metastable ( ^166m^Ho, T _1/2 _≈ 1200 year) with a low-background gamma-counter (Tobor, Nuclear Chicago, Chicago, IL, USA) as previously described in one of the preclinical studies by Zielhuis *et al*. [19].

### Primary objective

The primary objective of this study is to establish the safety and toxicity profile of treatment with ^166^Ho-PLLA-MS. This profile will be established using the CTCAE v3.0 methodology and will be used to determine the maximum tolerated radiation dose. Any of the following events which are considered possibly or probably related to the administration of ^166^Ho-PLLA-MS will be considered a serious adverse event during the 12 weeks follow-up period:

• Grade 3-4 neutropenic infection (absolute neutrophil count < 1.0 × 10 ^9^/L) with fever > 38.3°C,

• Grade 4 neutropenia lasting > 7 days,

• Grade 4 thrombocytopenia (platelet count < 25.0 ×10 ^9^/L),

• Grade 3 thrombocytopenia lasting for > 7 days,

• Any other grade 3 or 4 toxicity (excluding expected AST/SGOT, ALT/SGPT elevation, elevated bilirubin and lymphopenia) possibly related to study device, using CTCAE v3.0.

• Any life threatening event possibly related to the study device: events as a consequence of inadvertent delivery of ^166^Ho-PLLA-MS into non-target organs like the lung (radiation pneumonitis), the stomach and duodenum (gastric/duodenal ulcer or perforation), the pancreas (radiation pancreatitis), and liver toxicity due to an excessive radiation dose ("radiation induced liver disease" (RILD) [10]).

The haematological and biochemical adverse events as well as RILD will be considered dose limiting toxicity.

### Secondary objectives

Secondary objectives are to evaluate tumour response, performance status, biodistribution, quality of life and to compare the accuracy of the ^99m^Tc-MAA scout dose with a safety dose of ^166^Ho-PLLA-MS, in predicting microsphere distribution of the treatment dose. Tumour response will be quantified using CT of the liver scored according to Response Evaluation Criteria in Solid Tumours guidelines (RECIST 1.1) [27]. Tumour viability will be assessed by PET, depending on tumour type. In addition, the antitumoral effect will be assessed by relevant tumour markers responses if applicable (i.e. carcinoembryonic antigen (CEA) in colorectal carcinoma and chromogranin A (CgA) for neuroendocrine tumours). Biodistribution is assessed using quantitative SPECT and MRI. Urine and blood samples will be screened for presence of ^166^Ho-PLLA-MS or fragments of ^166^Ho-PLLA-MS. Performance status is assessed using WHO performance status criteria. Quality of life (QoL) is evaluated using the EORTC questionnaire QLQ-C30 with colorectal liver metastases module QLQ-LMC21. Finally, the accuracy of the ^166^Ho-PLLA-MS safety dose in predicting the distribution of the treatment dose is compared with the accuracy of the ^99m^Tc-MAA. Quantitative SPECT analysis will be performed using the scatter correction method described by De Wit *et al*. [14].

### Safety profile

From the literature on ^90^Y-RE, it is known that several treatment related effects can occur in radioembolization. As long as the patient is treated with the correct technique, which includes that no excessive radiation dose be delivered to any organ, the common adverse events after receiving radioactive microspheres are fever, abdominal pain, nausea, vomiting, diarrhoea and fatigue (i.e. postembolization syndrome) [10,28-30]. These effects are in general self-limiting within 1 to 2 weeks, and may be up to grade 3 or 4 (CTCAE v3.0) without direct clinical relevance. Based on the preclinical studies, a similar safety profile is expected for ^166^Ho-RE [22,23].

### Escape medication

Patients will receive oral analgesics (paracetamol up to 4000 mg/24 h) for relief of fever and pain after the administration of microspheres. To reduce nausea and vomiting, patients will receive anti-emetics (ondansetron up to 3 dd 8 mg) during the first 24 hours after administration of the treatment dose. In the case of persisting nausea, metoclopramid (up to 300 mg/24 h) will be used. Patients suffering from diarrhoea will receive loperamide (up to 16 mg/24 h). The vascular contrast agent jodixanol (Visipaque ^®^) may cause renal insufficiency in poorly hydrated patients. All patients will therefore be hydrated. This consists of 1.5 l NaCl 0.9% both prior to and post angiography. Inadvertent delivery of microspheres into organs such as the lungs, stomach, duodenum, pancreas, and gallbladder is associated with serious side effects. To reduce toxicity of the radioactive microspheres in patients with excessive extrahepatic deposition of ^166^Ho-PLLA-MS, the cytoprotective agent amifostine (Ethyol ^®^, up to 200 mg/m ^2 ^for 7 days) may be administered intravenously.

### Statistical considerations

Descriptive statistics (n, mean, standard deviation, median, minimum and maximum) will be calculated for each quantitative variable; frequency counts by category will be made for each qualitative variable. Interim analysis will be performed after every 3 patients. Inclusion of patients in the next cohort will be performed if the Independent Data Monitoring Committee (IDMC) has scrutinized the toxicity data and given permission to proceed.

Two sets of study data will be evaluated: the primary objective will be evaluated in the full analysis set (FAS). The FAS is defined as the set of data generated from the included patients who received at least the safety dose. The secondary objectives will be evaluated in both FAS and per-protocol set (PPS). The PPS is defined as the set of data generated from the included patients who complied with the protocol.

### Monitoring

The IDMC will perform a safety review after each series of treatments of three consecutive patients. The IDMC members have no conflict of interest with the sponsor because they are not involved in the study, nor are they receiving funds. The IDMC will work according to standard operating procedures and will receive reports on a regular basis on all toxicity CTCAE ≥ grade 3 reported for this trial. Recruitment will not be interrupted unless otherwise requested by the chairman of the IDMC. The responsibilities of the IDMC include:

• minimize the exposure of patients to an unsafe therapy or dose

• make recommendations for changes in study processes where appropriate

• endorse continuation of the study

• inform the institutional IEC in the case of toxicity CTCAE ≥ grade 3 and/or when the well-being of the subjects is jeopardized

### Ethical considerations

The study will be conducted according to the principles of the Declaration of Helsinki (version 9.10.2004) and in accordance with the Medical Research Involving Human Patients Act (WMO), the requirements of International Conference on Harmonization - Good Clinical Practice. The study protocol has been approved by the IEC and by the institutional Radiation Protection Committee.

## Discussion

The HEPAR trial is a phase I study to evaluate the safety and toxicity profile of ^166^Ho radioembolization. Secondary endpoints are tumour response, biodistribution assessment, performance status, quality of life and comparison of the biodistributions of the ^99m^Tc-MAA scout dose and the ^166^Ho-PLLA-MS safety dose.

With regard to the method of administration, viz. through a catheter placed in the hepatic artery, the in-vivo characteristics (no significant release of radionuclide), and the mechanism of action (local irradiation of the tumour), ^166^Ho-PLLA-MS constitute a device analogous to the ^90^Y microspheres, which are currently applied clinically. ^166^Ho-PLLA-MS only differ in the radioisotope and the device matrix that are used. In a toxicity study in pigs on ^166^Ho-RE, it has been demonstrated that (healthy) pigs can withstand extremely high liver absorbed doses, at least up to 160 Gy [23]. During these animal experiments, only very mild side effects were seen: slight and transitory inappetence and somnolence, which may well have been associated with the anaesthetic and analgesic agents that had been given and not necessarily with the microsphere administration. It is plausible that this low toxicity profile is caused by the inhomogeneous distribution of ^166^Ho within the liver after intra-arterial injection, as was observed on MRI and SPECT images. The current study will investigate whether a similar distribution pattern can also be observed in human subjects and whether this inhomogeneous distribution is concentrated around the tumour sites.

Hepatic arterial injection with ^99m^Tc-MAA and subsequent scintigraphic imaging is widely used to predict the biodistribution of ^90^Y microspheres, prior to the actual radioembolization procedure. Its accuracy can however be disputed. In our centre, we have observed that patients with a borderline lung shunt fraction of 10% to 19%, as calculated using the ^99m^Tc-MAA images (approximately 24% of all patients, all of whom were instilled a by 50% reduced amount of radioactivity), had no signs of lung shunting on post- ^90^Y-RE Bremsstrahlung images. In these cases, it seems that the ^99m^Tc-MAA-scan had false-positively predicted extrahepatic spread. This may be explained by the fact that ^99m^Tc-MAA differs in many aspects from the microspheres that are used. Shape, size, density, in-vivo half-life, and number of ^99m^Tc-MAA particles do not resemble the microspheres in any way [13,31]. In addition, free technetium that is released from the MAA particles can disturb the (correct) assessment of extrahepatic spread. We hypothesize that a small safety dose with low-activity ^166^Ho-PLLA-MS will be a more accurate predictor of distribution than ^99m^Tc-MAA. The unique characteristics of ^166^Ho-microspheres, in theory, allow a more accurate prediction of the distribution with the use of scintigraphy and MRI. In this study, we chose to perform both an injection with ^99m^Tc-MAA and administration of a safety dose of ^166^Ho-PLLA-MS. The respective distributions of the ^99m^Tc-MAA and the ^166^Ho-PLLA-MS safety dose will be compared with the distribution of the treatment dose of ^166^Ho-PLLA-MS by quantitative analysis of the scintigraphic images.

Both commercially available ^90^Y-MS products are approved by the Food and Drug Administration (FDA) and European Medicines Agency as a medical device and not as a drug. Radioactive microspheres are a medical device since these implants do not achieve any of their primary intended purposes through chemical action within or on the body and are not dependent upon being metabolized for the achievement of their primary intended purpose. In accordance with the definition of a medical device by the FDA and in analogy with the ^90^Y-MS, we consider the ^166^Ho-PLLA-MS to be a medical device [32]. The Dutch medicine evaluation board has discussed this issue (13 July 2007) and has concluded that the microspheres are indeed to be considered as a medical device.

One important issue concerning the resin-based SIR-Spheres ^® ^is the relatively high number of particles instilled (>1,000 mg), since this may sometimes be associated with macroscopic embolization as observed during the fluoroscopic guidance [28,33]. Several authors have reported stasis of flow during administration of resin microspheres and were forced to end the procedure prematurely because of the risk of backflow, hence extrahepatic deposition of a part of the dosage [28,34,35]. The specific activity of the ^166^Ho-PLLA-MS is considerably higher than that of the resin microspheres (≤450 and 50 Bq/microspheres, respectively). However, in order to obtain an equivalent absorbed dose, the total amount of radioactivity of the administered microspheres in ^166^Ho radioembolization needs to be 3 times higher than in ^90^Y radioembolization, due to the shorter physical half-life of ^166^Ho. Even so, compared with the resin ^90^Y microspheres, in ^166^Ho radioembolization considerably less microspheres (≤600 mg) are used to obtain an equivalent radiation dose, resulting in a lower risk of stasis or backflow during administration [9,29]. A further issue is that ^90^Y microspheres can not be visualized under fluoroscopy during injection. Manufacturers of resin ^90^Y microspheres state that their microspheres are to be administered with water for injection alternated with non-ionogenic contrast [36]. As a result, the operating physician cannot detect stasis or backflow of microspheres until he has switched from injecting microspheres to injecting the contrast agent. Holmium microspheres, on the contrary, are administered in a mixture of 50% saline and 50% non-ionogenic contrast under constant fluoroscopic imaging, which ensures constant control over the microspheres during injection [37]. However, continuous fluoroscopic imaging during microsphere administration may comprise an increased radiation dose delivered to the patient, specifically the abdominal skin, during the procedure. 	

If this phase I trial provides sufficient data to prove that ^166^Ho-PLLA-RE has an acceptable safety and toxicity profile, further studies will be needed. The next step will be an efficacy study in a larger number of patients. The primary endpoints of that study will be tumour response and survival.

## Appendix 1 - Eligibility criteria for ^166^Ho-RE

### Inclusion criteria

• Signed informed consent letter

• Age >18 years

• Liver-dominant metastases without standard treatment options. Liver-dominant disease is defined as the diameter of all metastases in the liver to be more than 200% of the sum of the diameters of all soft tissue lesions outside the liver.

• Life expectancy of ≥12 weeks

• World Health Organisation (WHO) Performance status 0-2

• ≥1 measurable lesions of ≥10 mm in the longest diameter by spiral computed tomography (CT) (5 mm slice thickness)

• Negative pregnancy test for women

### Exclusion criteria

• Brain metastases or spinal cord compression, unless irradiated at least 4 weeks prior to the date of the experimental treatment, and stable without steroid treatment for at least 1 week

• Radiation therapy within the last 4 weeks before study enrolment

• Patient has received chemotherapy within 4 weeks prior to enrolment

• Major surgery within 4 weeks, or incompletely healed surgical incision before enrolment

• Any unresolved toxicity greater than National Cancer Institute (NCI), Common Terminology Criteria for Adverse Events (CTCAE version 3.0)[26] grade 2 from previous anti-cancer therapy

• Alanine aminotransferase (ALT), aspartate aminotransferase (AST), or alkaline phosphatase (ALP) >5× Upper Limit of Normal (ULN), serum bilirubin >1.5× ULN or serum creatinine >185 µmol/L

• Leukocytes <4.0 10 ^9^/l and/or platelet count <150 10 ^9^/l

• Significant cardiac event (e.g. myocardial infarction, superior vena cava (SVC) syndrome, New York Heart Association (NYHA) classification of heart disease ≥2 within 3 months before entry, or presence of cardiac disease that, in the opinion of the investigator, increases the risk of ventricular arrhythmia

• Pregnancy or breast feeding

• Comorbidity with a grave prognosis (estimated survival <3 months) and/or worse than the basic disease for which the patients will be included in the study

• Abnormalities of the bile ducts (such as stents) with an increased chance of infection

• Diseases with an increased chance of liver toxicity, such as primary biliary cirrhosis or xeroderma pigmentosum

• Patients who are declared incompetent or have a psychiatric disorder that makes a comprehensive judgement impossible, such as psychosis, hallucinations and/or depression

• Previous enrolment in the present study or previous treatment with radioembolization

• Treatment with an investigational agent within 42 days prior to enrolment

• Female patients who are not using an acceptable method of contraception or are less than 1 year postmenopausal or surgically sterile during their participation in this study (from the time the consent form is signed) to prevent pregnancy

• Male patients who are not surgically sterile or do not use an acceptable method of contraception during their participation in this study to prevent pregnancy in a partner

• Evidence of portal hypertension, splenomegaly or ascites

• Body weight >150 kg

• Active hepatitis (B and/or C)

• Liver weight >3 kg (determined by software using CT data)

• Allergy for intravenous contrast agent used (Visipaque ^®^)

• General MRI contra-indications (severe claustrophobia, metal implants, implanted pacemaker and/or neurostimulators)

• Patients who have arterial variations that will not allow whole liver treatment by a single administration via the hepatic artery

## Competing interests

The authors declare that they have no competing interests.

## Authors' contributions

All authors contributed to the study design. BZ is the study's principal investigator. The manuscript was written by MS, JN, MvdB, ML, MV, and AvhS. All authors revised the manuscript and approved the final version of the manuscript.
